# Utility of Minimally Invasive Cardiovascular Monitoring in the High-Risk Patient Undergoing Radical Cystoprostatectomy: A Case Report

**DOI:** 10.7759/cureus.10936

**Published:** 2020-10-14

**Authors:** Nasrin N Aldawoodi, Jamie P Hoffman, Allan R Escher

**Affiliations:** 1 Anesthesiology, H. Lee Moffitt Cancer Center and Research Institute, Tampa, USA; 2 Anesthesiology/Pain Medicine, H. Lee Moffitt Cancer Center and Research Institute, Tampa, USA

**Keywords:** mitral stenosis, squamous cell carcinoma, pulmonary hypertension, goal-directed, cystoprostatectomy, ileal conduit, tee, minimally invasive, svv, minimally invasive cardiovascular monitoring

## Abstract

Non-cardiac surgery in a high-risk patient with severe mitral stenosis (MS) and severe pulmonary hypertension (PH) presents a significant anesthetic challenge. Guidelines recommend using advanced hemodynamic monitors for specific cardiovascular goals. The gold standard for intraoperative monitoring in these cases is the pulmonary artery catheter (PAC) and transesophageal echocardiography (TEE). This case discusses the successful management of a severe MS patient undergoing cystoprostatectomy using a minimally invasive cardiovascular monitor (MICM) incorporating several hemodynamic parameters.

## Introduction

The etiology of mitral stenosis (MS) includes rheumatic fever and degenerative mitral valve (MV) disease [1]. Per American Heart Association/American College of Cardiology (AHA/ACC) guidelines, asymptomatic patients with severe MS may undergo non-cardiac surgery with appropriate management [2]. Goals include avoidance of tachycardia, maintenance of sinus rhythm and afterload, and judicious preload augmentation [3]. Severe pulmonary hypertension (PH) warrants the avoidance of hypoxia, hypercarbia, and acidosis. Advanced hemodynamic monitoring is indicated for these patients. The use of a minimally invasive cardiovascular monitor (MICM) can be a critical component for the management of high-risk patients having extensive surgical procedures.

## Case presentation

A 78-year-old man with squamous cell bladder carcinoma and high-grade prostate cancer presented for radical cystoprostatectomy, lymph node dissection, and ileal conduit. His medical history included hypertension (HTN), transient ischemic attack (TIA) x 1, and gastroesophageal reflux disease (GERD). In the preanesthesia testing clinic, a 2/6 diastolic murmur was auscultated. An externally performed transthoracic echocardiogram (TTE) showed an ejection fraction (EF) of 65%-70%, dilated right ventricle (RV) with normal systolic function, severely dilated left atrium, heavily calcified mitral annulus and valve leaflets, and severe MS (area 1.47 cm2, mean gradient 14 mmHg). There was concurrent mild aortic stenosis (valve area 1.83 cm2, mean gradient 19 mmHg), mild mitral regurgitation (MR), moderate tricuspid regurgitation (TR), and a right ventricular systolic pressure of 67 mmHg. Given his aggressive cancers and lack of symptoms, a decision was made to proceed to the operating room (OR) without further testing or intervention. Initial preoperative vitals were blood pressure (BP) 134/77 mmHg, heart rate (HR) 85 beats per minutes (bpm), and oxygen saturation (SpO2) 94% on room air. His electrocardiogram (EKG) showed sinus rhythm (SR) with first-degree atrioventricular (AV) block and right bundle branch block (RBBB).

In the OR, standard American Society of Anesthesiologists (ASA) monitors were applied. Defibrillator pads were placed, and amiodarone was available. A pre-induction arterial line was placed with 1% subcutaneous lidocaine. After preoxygenation, the patient was induced and intubated with intravenous (IV) lidocaine 60 mg, etomidate 20 mg, succinylcholine 100 mg, and fentanyl 250 mcg in rapid succession. Esmolol was given for a total of 40 mg IV to maintain a heart rate less than 70 bpm during laryngoscopy. The patient was placed on a low tidal volume strategy 6 mL/kg, relatively high respiratory rate, an inspiratory to expiratory (I:E) ratio of 1:2, and a positive end-expiratory pressure (PEEP) of 4. The goal was to keep intrathoracic pressures low to avoid increasing RV afterload and pulmonary vascular resistance (PVR) and decreasing RV preload. An arterial blood gas was obtained 30 minutes after intubation to calibrate the end tidal CO2 to the pCO2 and check the pH. Norepinephrine and epinephrine bolus syringes and infusions were prepared. An eight-French (FR), 16-cm double lumen central venous line was inserted into the right internal jugular vein using ultrasound. The arterial and central lines were connected to an MICM device.

**Figure 1:**
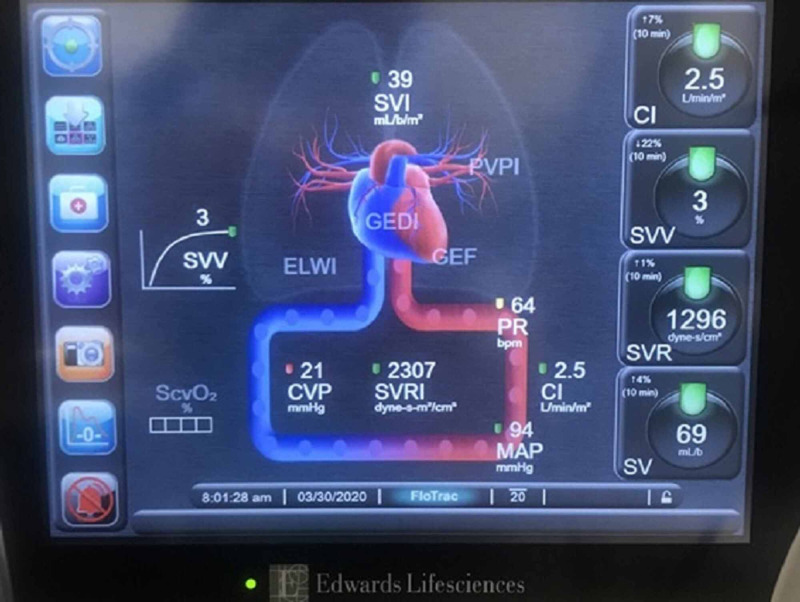
Intraoperative minimally invasive cardiac monitoring device with hemodynamic parameters. SVV, stroke volume variation; SV, stroke volume; SVI, stroke volume index; CVP, central venous pressure; CI, cardiac index; PR, pulse rate; MAP, mean arterial pressure; SVR, systemic vascular resistance; GEDI, global-end diastolic volume index; PVPI, pulmonary vascular permeability index; ELWI, extravascular lung water index; GEF, global ejection fraction; SVRI, systemic vascular resistance index.

Initial readings showed central venous pressure (CVP) of 21 mmHg, cardiac index (CI) of 2.5 L/min/m2, stroke volume variation (SVV) of 3, systemic vascular resistance (SVR) of 1296 dynes-s/cm5, and mean arterial pressure (MAP) of 94 mmHg (Figure 1).

Goals were to keep HR < 70 bpm using fentanyl and esmolol and to maintain afterload with MAP > 70 mmHg, which was 20% of patient’s baseline MAP. If MAP dropped below 70 mmHg, and SVV was less than 13%, boluses of 100 mcg IV phenylephrine were given due to the favorable reflex bradycardia. Boluses of norepinephrine or ephedrine were ready in case of HR < 50 bpm or a lack of response to phenylephrine. Epinephrine was reserved in case of hemodynamic collapse refractory to other vasopressors.

**Figure 2:**
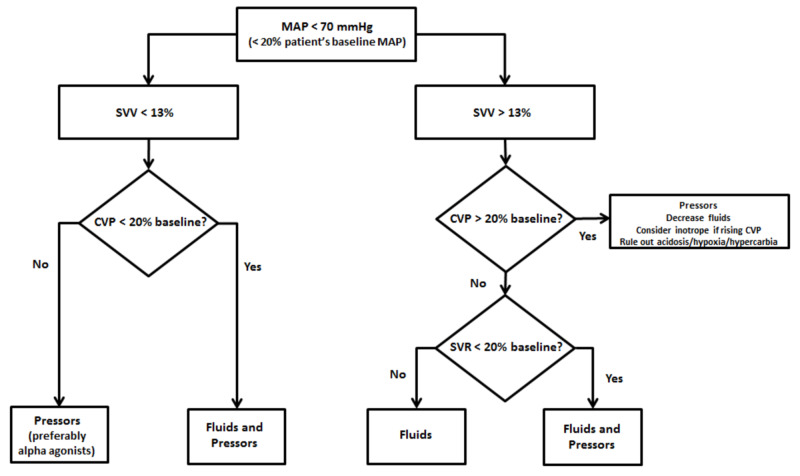
Intraoperative fluid and vasopressor management algorithm for mitral stenosis and pulmonary hypertension using minimally invasive cardiac monitoring. CVP, central venous pressure; MAP, mean arterial pressure; SVR, systemic vascular resistance; SVV, stroke volume variation.

If SVV was > 13%, a bolus of 250 mL of 5% albumin IV was given while watching the CVP trend. The goal was normovolemia, with an SVV < 13% and CVP within 20% in each direction of patient’s baseline CVP. Specifically, a rise in CVP of >20% of baseline values was interpreted as possible RV overload and risk of failure. Hence, if CVP continued to rise, the plan was to hold fluids and start an RV inotrope such as an epinephrine. At that point, arterial blood gas would be checked to ensure patient was not acidotic or hypoxic. Crystalloid administration was limited in favor of albumin and packed red blood cells (pRBCs) depending on surgical blood loss. CVP and SVR parameters were further used to guide fluid and pressor use as follows: If SVV was > 13% but SVR was < 20% of baseline or if SVV was < 13% but CVP dropped > 20% baseline, a combination of albumin and phenylephrine was given (Figure 2). 

Urine output was not available during the case as the ureters were transected.

The surgery lasted four hours and 45 minutes. Blood loss was 300 mL, and 1700 mL of crystalloid and 500 mL of 5% albumin were given. To avoid tachycardia on emergence, 30 mg IV esmolol was given. Suggamadex was used for reversal of relaxation. Patient remained hemodynamically stable throughout and recovered uneventfully on the floor with telemetry monitoring. He was discharged home on post-operative day 6.

## Discussion

While MS occurs in only 0.02%-0.2% of the population, degenerative calcific MS is increasing in prevalence [1,3]. Patients with asymptomatic severe MS may proceed with elective non-cardiac surgery if the valve is not favorable for percutaneous balloon commissurotomy [2]. In this case, a radical cystoprostatectomy is an elevated risk procedure, and the patient had not been evaluated for ischemic heart disease or ballooning of the valve. However, the decision was made to proceed with surgery as he had excellent exercise capacity and high-grade cancers.

A key hemodynamic goal included maintaining diastolic left ventricular (LV) filling to maintain cardiac output. This was accomplished by avoiding tachycardia and hypovolemia as well as maintaining SR and the atrial kick. These patients are predisposed to atrial fibrillation due to left atrial enlargement. Afterload was maintained for coronary perfusion and preventing ischemia. An epidural, while not absolutely contraindicated, was not placed to avoid precipitous drops in preload and afterload. The concurrent severe PH from increased left atrial pressures could precipitate right heart failure. Hence, to avoid increasing pulmonary artery (PA) and RV pressures, care was taken to avoid hypercarbia, hypoxia, acidosis, and excessive preload. Increased intrathoracic pressure from positive pressure ventilation and PEEP can be deleterious to RV function, thus necessitating low PEEP and tidal volumes with higher respiratory rates. Inotropic agents were available in case of suspected RV failure [3]. 

For intraoperative monitoring in MS patients with severe PH, guidelines recommend TEE or a PAC to assess volume, biventricular function, and cardiac output [2]. However, both modalities are invasive, associated with complications and questionably improve outcomes [4,5,6]. Studies have found PA occlusion pressure not to be a useful predictor of ventricular preload with respect to optimizing cardiac performance [7]. Indeed, PAC usage has declined with minimally invasive cardiac monitoring devices increasingly being used [4]. 

In this case, a MICM device was used to monitor preload, CO, and afterload by trending SVV, CI, SV, and SVR using an arterial line and central line. The reliability of non-invasive monitoring has been explored in recent literature. One study comparing MICM to a PAC found that SVV correlated better with right ventricular end diastolic volume index than PA diastolic pressure, suggesting it was an accurate preload monitor [8]. Other studies found that cardiac output and SVV by minimally invasive cardiac monitoring devices correlated well with TEE findings [4,9]. In fact, several algorithms have been developed using SVV and other non-invasive parameters to guide fluid optimization in high-risk patients [10].

However, few studies address the efficacy of non-invasive hemodynamic monitors in patients with significant valvulopathies. Case reports describe using the MICM for management of severe aortic stenosis during hip surgery and severe MR during cerebral aneurysm surgery [11,12]. However, recent randomized trials using these devices on patients undergoing large abdominal surgeries specifically excluded patients with moderate to severe valve disease [13]. 

This case report describes a unique MICM algorithm incorporating MAP, SVV, SVR, and CVP for intraoperative management of severe MS with severe PH. Of note, CVP has been shown to poorly predict responsiveness to a fluid challenge and should not be used alone to guide therapy [14]. However, obtaining a baseline value and trend is helpful to assess RV filling pressure for signs of RV overload [15]. 

## Conclusions

Minimally invasive cardiac monitoring devices have the potential to lower complications and may serve as a less invasive alternative for intraoperative hemodynamic monitoring. The high-risk patient population may especially benefit from such an approach to non-cardiac surgeries. Further clinical studies are needed to elucidate the utility of MICM in comparison to the PA catheter in similar high-risk patients.
